# Probabilidade de morte prematura por doenças crônicas não transmissíveis: desafios para o alcance das metas dos Objetivos do Desenvolvimento Sustentável no Brasil e Unidades Federadas

**DOI:** 10.1590/0102-311XPT038825

**Published:** 2025-10-24

**Authors:** Deborah Carvalho Malta, Érika Carvalho de Aquino, Laís Santos de Magalhães Cardoso, Guilherme Augusto Veloso, Ana Maria Nogales Vasconcelos, Letícia de Oliveira Cardoso, Regina Tomie Ivata Bernal, Juliana Bottoni de Souza, Filipe Malta dos Santos, Mohsen Naghavi, Maurício Lima Barreto

**Affiliations:** 1 Universidade Federal de Minas Gerais, Belo Horizonte, Brasil.; 2 Centro de Estudos Estratégicos, Fundação Oswaldo Cruz, Rio de Janeiro, Brasil.; 3 Centro de Integração de Dados e Conhecimentos para Saúde, Fundação Oswaldo Cruz, Salvador, Brasil.; 4 Universidade Federal Fluminense, Niterói, Brasil.; 5 Universidade de Brasília, Brasília, Brasil.; 6 Escola Nacional de Saúde Pública Sergio Arouca, Fundação Oswaldo Cruz, Rio de Janeiro, Brasil.; 7 Faculdade Ciências Médicas de Minas Gerais, Belo Horizonte, Brasil.; 8 Institute for Health Metrics and Evaluation, University of Washington, Seattle, USA.

**Keywords:** Doenças Não Transmissíveis, Mortalidade Prematura, Desenvolvimento Sustentável, Estudos de Séries Temporais, Noncommunicable Diseases, Premature Mortality, Sustainable Development, Time Series Studies, Enfermedades No Transmisibles, Mortalidad Prematura, Desarrollo Sostenible, Estudios de Series Temporales

## Abstract

As doenças crônicas não transmissíveis (DCNT) são a principal causa de morbimortalidade no Brasil. O estudo visa verificar se a meta de redução das DCNT dos Objetivos de Desenvolvimento Sustentável (ODS) até 2030 será alcançada por meio da análise das tendências da probabilidade incondicional de morte prematura entre 1990 e 2021 no Brasil e nas 27 Unidades Federadas. Realizou-se estudo de série temporal sobre a probabilidade de morte prematura (30-69 anos) por DCNT (doenças cardiovasculares, neoplasias, doenças respiratórias crônicas e diabetes mellitus), com base nos dados do *Global Burden of Disease Study* de 2021. Foram utilizados modelos de regressão por pontos de inflexão (*joinpoint*) para estimar tendências, além de projeções até 2030 por meio do modelo de Holt. As desigualdades regionais foram avaliadas com base nos quintis do índice sociodemográfico (SDI, acrônimo em inglês). A probabilidade de morte prematura por DCNT reduziu de 0,233 (1990) para 0,152 (2021) (AAPC = -1,3; p < 0,001), com declínio em todos os quintis do SDI. A mortalidade foi consistentemente maior entre os homens. As projeções indicam que a meta de redução de 1/3 até 2030 provavelmente não será alcançada, especialmente nos quintis de menor SDI, com variações segundo o sexo. Apesar da tendência de queda, persistem desigualdades regionais e sociais. Melhorias no acesso à saúde e em políticas públicas contribuíram para o declínio observado, mas desafios permanecem, como o enfraquecimento das políticas de controle de fatores de risco, a influência dos determinantes comerciais da saúde e os efeitos da pandemia de COVID-19.

## Abstract (en)

Noncommunicable diseases (NCDs) are the leading causes of morbidity and mortality in Brazil. The aim of the present study was to determine whether the reduction in the occurrence of NCDs by 2030, which is one of the Sustainable Development Goals (SDGs), will be achieved through an analysis of trends in the unconditional probability of premature deaths between 1990 and 2021 in Brazil and its 27 states. A time-series study was conducted on the probability of premature death (30-69 years of age) due to NCDs (cardiovascular diseases, neoplasms, chronic respiratory diseases, and diabetes mellitus) based on data from the 2021 *Global Burden of Disease Study*. Joinpoint regression models were used to estimate trends and projections up to 2030 using Holt’s model. Regional inequalities were assessed based on quintiles of the sociodemographic index (SDI). The probability of premature death due to NCDs reduced from 0.233 (1990) to 0.152 (2021) (average annual percent change = -1.3; p < 0.001), with a decline in all SDI quintiles. Mortality was consistently higher among men. Projections indicate that the target of a one-third reduction by 2030 will likely not be achieved, especially in the lowest SDI quintiles, with variations according to sex. Despite the downward trend, regional and social inequalities persist. Improvements in access to health care and public policies contributed to the decline, but challenges remain, such as the weakening of risk factor control policies, the influence of commercial determinants of health, and the effects of the COVID-19 pandemic.

## Abstract (es)

Las enfermedades crónicas no transmisibles (ECNT) son la principal causa de morbimortalidad en Brasil. Este estudio tiene como objetivo estimar si se logra la meta de los ODS de reducir las ECNT para 2030 con base en el análisis de las tendencias en la probabilidad incondicional de muerte prematura entre 1990 y 2021 en Brasil y sus 27 Unidades Federadas. Se realizó un estudio de series temporales sobre la probabilidad de muerte prematura (30-69 años de edad) por ECNT (enfermedades cardiovasculares, neoplasias, enfermedades respiratorias crónicas y diabetes mellitus), basado en datos de GBD 2021. Se utilizaron modelos de regresión por puntos de inflexión (*joinpoint*) para estimar tendencias, además de proyecciones para 2030 mediante el modelo Holt. Las desigualdades regionales se evaluaron en función de los quintiles de SDI. La probabilidad de muerte prematura por ENT tuvo una reducción de 0,233 (1990) a 0,152 (2021) (AAPC = -1,3; p < 0,001), con una disminución en todos los quintiles de SDI. La mortalidad fue consistentemente más alta entre los hombres. Las estimaciones indican que la meta de reducción de 1/3 para 2030 probablemente no se alcanzará, especialmente en los quintiles de SDI más bajos, con variaciones según el sexo. A pesar de la tendencia a la baja, persisten las desigualdades regionales y sociales. Las mejoras en el acceso a la salud y en las políticas públicas contribuyeron a la observada reducción, aunque persisten desafíos, como el debilitamiento de las políticas de control de factores de riesgo, la influencia de los determinantes comerciales de la salud y los efectos de la pandemia de la COVID-19.

## Introdução

As doenças crônicas não transmissíveis (DCNT) representam a maior causa de morbimortalidade no Brasil e no mundo, além de resultarem em mortes prematuras, incapacidades, perda da qualidade de vida e importantes impactos sociais ^1^ ^,^ ^2^. Contribuem, ainda, para o aumento das internações hospitalares e sobrecarga dos sistemas de saúde ^3^. Estima-se que as DCNT sejam responsáveis por 75% da mortalidade geral (equivalente a 41 milhões de óbitos) no mundo, sendo 15 milhões de mortes prematuras ^4^.

Os quatro principais grupos de DCNT (doenças cardiovasculares, câncer, doenças respiratórias crônicas e diabetes) compartilham fatores de riscos comportamentais modificáveis, como o tabagismo, consumo abusivo de bebidas alcoólicas, inatividade física e alimentação inadequada ^5^ ^,^ ^6^. Estes, portanto, devem ser prioridade nas ações e políticas públicas que envolvem a promoção da saúde, ações regulatórias e de prevenção desses fatores de risco. Os fatores de risco metabólicos (obesidade, glicemia elevada, hipertensão arterial, colesterol elevado e outros) têm importante papel na causalidade das DCNT ^7^.

Os determinantes sociais e ambientais como renda, educação e local de moradia também contribuem para o aumento e a gravidade das DCNT e da morbimortalidade associada a estas ^8^. Indivíduos de baixa renda, socialmente desfavorecidos ou marginalizados têm maior exposição aos fatores de risco e acesso diminuído aos serviços de saúde, à alimentação saudável e a ambientes saudáveis ^8^ ^,^ ^9^. Além disso, esses indivíduos podem ter sua condição de pobreza exacerbada pelas DCNT devido a possíveis incapacidades decorrentes destas, maiores despesas familiares com o tratamento da doença e maior demanda por serviços de assistência à saúde ^10^. Destaca-se, ainda, o papel dos determinantes comerciais ligados às dinâmicas de mercados nacionais e transnacionais, que podem dificultar a implantação de políticas públicas eficazes no enfrentamento ao tabagismo e ao consumo de álcool e de alimentos ultraprocessados. As práticas corporativas ou interesses comerciais podem comprometer a saúde e o meio ambiente por meio, por exemplo, da interferência no parlamento, fragilizando os governos e estados na condução de medidas regulatórias para redução destes fatores de risco ^11^.

Frente a esse grave problema de saúde pública, iniciativas globais foram implementadas no intuito de prevenir e controlar a carga das DCNT e seu impacto para os governos, sistemas de saúde, indivíduos, famílias e comunidades. Como marco global, destacam-se os Objetivos dos Desenvolvimento Sustentável (ODS), cujo objetivo 3 tem a finalidade de “*assegurar uma vida saudável e promover o bem-estar para todas e todos, em todas as idades*”. A meta 3.4, abrangida neste objetivo, consiste em “*até 2030, reduzir em um terço a mortalidade prematura por doenças não transmissíveis via prevenção e tratamento, e promover a saúde mental e o bem-estar*” ^12^ ^,^ ^13^. Destaca-se, ainda, que a meta de redução da mortalidade prematura por DCNT também está contida no Plano Global de Ações para prevenção e controle das DCNT, da Organização Mundial da Saúde (OMS) ^4^, além do Plano de Ações Estratégicas para o Enfrentamento das DCNT 2011-2022 ^14^ ^,^ ^15^ e na sua atualização, o Plano de Ações Estratégicas para o Enfrentamento das Doenças Crônicas e Agravos não Transmissíveis 2021-2030 ^16^.

É importante salientar que a pandemia de COVID-19 acarretou mudanças nos indicadores de saúde e, consequentemente, piorou os padrões de saúde da população, com aumento dos fatores de risco e redução dos fatores de proteção para as DCNT. Esse impacto foi ainda mais grave em países com situação de vulnerabilidade e desigualdade, e que tiveram cortes nas políticas sociais. Além disto, a pandemia também influenciou na capacidade da atenção integral dos serviços de saúde, com descontinuidade ou interrupção da assistência a pessoas em tratamento das DCNT e nos serviços de promoção da saúde ^17^ ^,^ ^18^, resultando em piora no controle dessas doenças e no excesso de mortalidade ^19^.

O monitoramento da distribuição, magnitude e tendência das DCNT e seus fatores de risco e de proteção, bem como da morbimortalidade, é essencial, especialmente face às mudanças no cenário político, econômico e de saúde que ocorrem no Brasil ^20^. Ademais, as metas de redução das DCNT devem ser objeto de contínuo monitoramento pelo país, uma vez que contribuem para reorientação dos serviços de saúde e do processo de trabalho e auxiliam na revisão de estratégias para a prevenção, enfrentamento e controle das DCNT ^21^.

Nesse sentido, o objetivo deste estudo foi verificar se a meta de redução das DCNT dos ODS até 2030 será alcançada por meio da análise das tendências da probabilidade incondicional de morte prematura, entre 1990 e 2021, no Brasil e nas 27 Unidades Federadas (UF).

## Métodos

Trata-se de uma análise de série temporal da probabilidade de morte prematura por DCNT no Brasil e suas 27 unidades federadas, de 1990 a 2021.

As DCNT foram classificadas de acordo com a Classificação Estatística Internacional de Doenças e Problemas Relacionados à Saúde, 10ª revisão (CID-10), com os seguintes códigos: doenças cardiovasculares (I00 a I99), doenças respiratórias (J30-J98), neoplasias (C00-C97) e diabetes mellitus (E10-E14). Considerou-se morte prematura aquela ocorrida dos 30 aos 69 anos de idade ^14^. 

### Probabilidade incondicional de morte prematura por DCNT

A probabilidade de morte foi aferida, segundo referência da OMS, calculando-se inicialmente a taxa de mortalidade específica por idade para cada faixa etária de 5 anos, segundo a metodologia: (a) transformar as taxas quinquenais em probabilidades de morte em cada faixa etária (taxa_y_ × 5)/(1 + taxa_y_ × 2,5); (b) em seguida, multiplicar os complementares dessas probabilidades de morte, que são as probabilidades de sobrevivência (1-probabilidade_y_); (c) e, por último, obter o complementar dessa multiplicação 1-produtório (1-probabilidade_y_), que é a probabilidade de morte no grupo etário 30-69 anos ^4^ ^,^ ^22^.

Este indicador é preconizado pela OMS e utiliza métodos de tábua de vida, permitindo o cálculo do risco de morte entre as idades exatas 30 e 69 anos por DCNT, na ausência de outras causas de morte. Este indicador foi escolhido para excluir o viés entre países e regiões ao longo do tempo devido a diferenças ou mudanças nas taxas de mortalidade para outras causas concorrentes e para controlar as diferenças na estrutura etária da população ^22^ ^,^ ^23^.

### Índice sociodemográfico

Para aferir a desigualdade entre as regiões, foi analisada a relação entre o índice sociodemográfico (*sociodemographic index* - SDI) e a probabilidade de morte por DCNT. O SDI é um indicador composto estimado para cada região, país e estado ^24^. 

A utilização do SDI como indicador sociodemográfico justifica-se por sua capacidade de refletir, de forma abrangente, o nível de desenvolvimento das UF, ao combinar informações sobre renda *per capita*, escolaridade média e taxa de fecundidade total ^24^. Esses três componentes são determinantes fundamentais da saúde e influenciam diretamente os padrões de morbimortalidade por DCNT. Assim, o SDI permite uma análise mais robusta das desigualdades regionais e contribui para a compreensão das disparidades na probabilidade de morte prematura ^25^.

**Figura 1:**
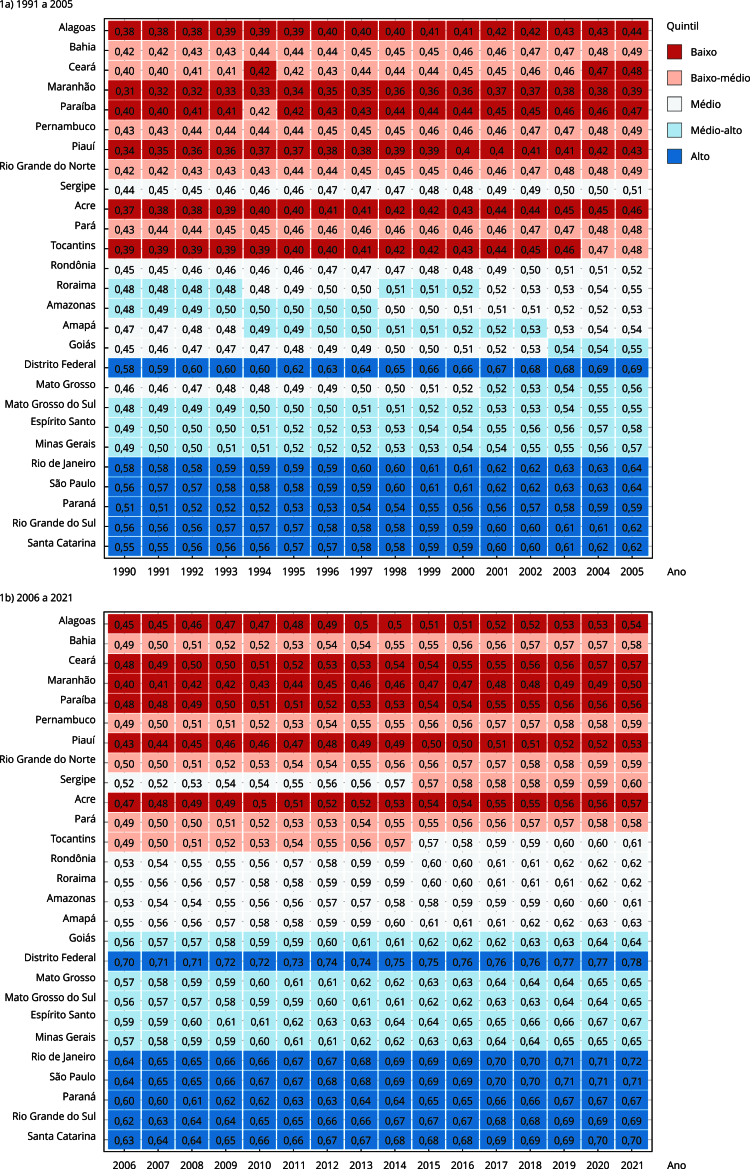
Índice sociodemográfico (SDI, acrônimo em inglês) das Unidades Federadas brasileiras (1990-2021).

Os valores de SDI variam de 0 a 1, sendo 0 o pior escore e 1 o melhor ^24^. Para cada ano, de 1990 a 2021, foram calculados os quintis do SDI considerando as UF, sendo possível classificá-las em cinco grupos. Os valores dos quintis de SDI sofreram variações ao longo do período de 1990 a 2021. No primeiro ano (1990), o SDI do quintil baixo variou entre 0,38 e 0,40, do médio-baixo de 0,40 a 0,43, do médio de 0,45 a 0,47, do médio-alto de 0,48 a 0,49 e do alto de 0,51 a 0,58. Em 2021, o SDI do quintil baixo variou entre 0,5 e 0,56, do médio-baixo de 0,58 a 0,60, do médio de 0,61 a 0,63, do médio-alto de 0,64 a 0,67 e do alto de 0,71 a 0,78 (Figura 1). Para cada quintil foi calculada a probabilidade de morte ao longo do tempo.

### Fontes de dados 

Este estudo utilizou a base de dados do estudo GBD 2021 do Instituto de Métricas e Avaliação em Saúde (IHME, acrônimo em inglês), disponível em https://vizhub.healthdata.org/gbd-compare/. As estimativas de mortalidade do IHME são obtidas a partir de dados do Sistema de Informações sobre Mortalidade (SIM) ^26^ do Ministério da Saúde, com ajuste para subregistro e códigos *garbage*, conforme algoritmos de redistribuição para causas básicas de morte segundo idade-sexo-ano ^27^ ^,^ ^28^. É considerada a população padrão global denominada *GBD World Standard Population* (GBD WSP) ^29^.

### Análises de séries temporais

Para analisar as tendências de probabilidade incondicional de morte prematura por DCNT, utilizou-se o modelo de regressão por pontos de inflexão (*joinpoint regression model*). A regressão de *joinpoint* permite identificar se existem diferentes padrões de tendência ao longo do tempo. Assume-se que a série temporal é composta por uma série de segmentos com diferentes inclinações, que são unidos por pontos de mudança. A técnica busca encontrar o número e a localização desses pontos de mudança, ajustando uma linha reta em cada segmento identificado ^30^ ^,^ ^31^. Ressalta-se que cada ponto de inflexão reflete uma alteração significativa na trajetória do indicador. Para mensurar essas mudanças, foi calculada a variação percentual anual (APC, acrônimo em inglês) para cada período identificado. Além disso, a variação percentual média anual (AAPC, acrônimo em inglês) foi utilizada para a série temporal completa. Considerou-se a presença de tendência estatisticamente significativa quando os valores de APC e AAPC foram estatisticamente diferentes de zero, considerando-se o valor de p ≤ 0,05.

Em função da meta dos ODS de redução de 30% da probabilidade incondicional de morte por DCNT entre 2015 e 2030, foi realizada a projeção do indicador de 2022 a 2030 para o Brasil pelos 05 (quintis) do SDI relativo aos 27 estados segundo sexo, aplicando-se o modelo de Suavização Exponencial de Holt (SEH) ^31^ aos dados observados (1990 a 2021). Este método é utilizado para realizar previsões em séries temporais que apresentam tendência, mas não apresentam sazonalidade. O modelo usa duas componentes principais: nível (a média da série temporal, que é ajustada a cada novo ponto de dados) e tendência (a direção da mudança na série, ou seja, a taxa de crescimento ou declínio). É possível realizar previsões ao combinar esses dois componentes, ajustando-os dinamicamente conforme novos dados são adicionados.

Adicionalmente, as projeções da probabilidade incondicional de morte prematura por DCNT para o período de 2022 a 2030 foram estratificadas por sexo, de forma a permitir a avaliação diferenciada do alcance da meta dos ODS entre homens e mulheres. O modelo de Holt foi aplicado de maneira independente para cada grupo populacional (masculino e feminino), considerando os dados observados de 1990 a 2021. Essa abordagem possibilitou a comparação das tendências projetadas segundo o sexo e o nível de desenvolvimento sociodemográfico (quintis de SDI).

Embora a meta pactuada pelos ODS estabeleça o ano de 2015 como marco inicial para a redução de um terço da mortalidade prematura por DCNT até 2030, optou-se por utilizar o período completo de 1990 a 2021 na análise da série temporal. Essa escolha se justifica pelo fato de que séries históricas mais extensas permitem maior robustez na identificação de tendências e pontos de inflexão por meio da regressão de *joinpoint*, além de fornecerem uma base empírica mais estável para a aplicação de modelos de projeção, como o de Holt, utilizado neste estudo ^32^. Ademais, a consideração de um intervalo temporal mais amplo possibilita compreender o contexto histórico das transições epidemiológicas e demográficas no Brasil, bem como avaliar a trajetória da mortalidade prematura por DCNT antes e após a pactuação da meta dos ODS, contribuindo para uma análise crítica da viabilidade de seu alcance.

As análises foram conduzidas no software R (http://www.r-project.org) e as figuras foram produzidas por meio do pacote *ggplot*.

Esta pesquisa foi conduzida em conformidade com a *Resolução nº 466*, de 12 de dezembro de 2012, do Conselho Nacional de Saúde (CNS), que estabelece diretrizes e normas regulamentadoras de pesquisas envolvendo seres humanos. O estudo foi aprovado pelo Comitê de Ética em Pesquisa com Seres Humanos da Universidade Federal de Minas Gerais (UFMG, parecer nº 3.258.076).

## Resultados

A Figura 1 aponta que os quintis de SDI mantiveram-se relativamente estáveis ao longo do tempo. Os estados brasileiros apresentam variação do SDI entre si e no decorrer do tempo. O menor valor para o indicador ocorreu em Alagoas, em 1991 (0,38). O maior ocorreu no Distrito Federal, em 2021. 

As UF abrangidas no quintil de baixo SDI (1): Alagoas, Maranhão, Paraíba, Piauí, Acre e Tocantins. Este último permanece no quintil de baixo SDI entre 1990 e 2003, e depois sobe para o quintil baixo-médio. O quintil baixo-médio é composto pelos seguintes estados: Bahia (1990-1993), Ceará (1994-2003), Pernambuco (1990-2021), Rio Grande do Norte (1990-2021), Pará (1990-2021), Tocantins (2004-2014) e Sergipe (2014-2021). O quintil médio é composto por: Sergipe (1990-2014), Tocantins (2015-2021), Rondônia (1994-1997), Roraima (2001-2021), Amazonas (1998-2021), Amapá (1998-2021), Goiás (1990-2022) e Mato Grosso (1990-2000). O quintil médio-alto foi composto por Roraima (1990-1993 e 2000-2003), Amazonas (1990-1997), Amapá (1994-2002), Goiás (2003-2021), Mato Grosso (2002-2021), Mato Grosso do Sul (1990-2021), Espírito Santo (1990-2021) e Minas Gerais (1990-2021). O quintil alto é composto pelo Distrito Federal, Rio de Janeiro, São Paulo, Paraná, Rio Grande do Sul e Santa Catarina. Todas as UF classificadas neste quintil se mantiveram no mesmo período de 1990 a 2021.

**Figura 2:**
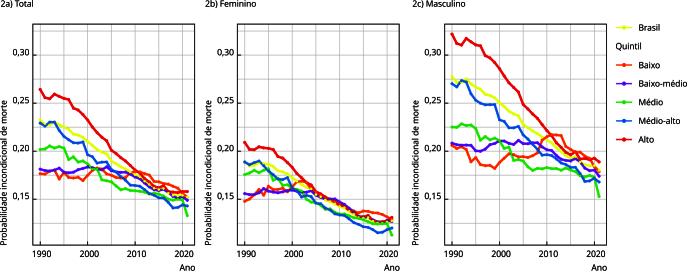
Probabilidade incondicional de morte prematura por doenças crônicas não transmissíveis segundo sexo no Brasil (1990-2021).

Observa-se declínio da probabilidade de morte prematura por DCNT no Brasil no período de 1990 a 2021, passando de 23,3% para 15,2% (AAPC = -1,3; p < 0,001). Além da população geral, a queda pôde ser observada em ambos os sexos (Figura 2). Houve redução na probabilidade de morte em todos os quintis de SDI: no quintil de baixo SDI, houve redução de 17,7% para 15,2% (AAPC = -0,5; p < 0,001), no quintil médio-baixo de 18,1% para 14,9% (AAPC = -0,6; p < 0,001), no médio de 20,2% para 13,3% (AAPC = -1,3; p < 0,001), no médio-alto de 22,9% para 14,3% (AAPC = -1,5; p < 0,001) e no alto de 26,4% para 15,8% (AAPC = -1,6; p < 0,001) (Tabela 1).

**Tabela 1:** Variação percentual média anual (AAPC, acrônimo em inglês) da probabilidade incondicional de morte prematura por doenças crônicas não transmissíveis, por sexo e quintil do índice sociodemográfico (SDI, acrônimo em inglês) no Brasil (1990-2021).

	Total				Feminino				Masculino			
	1990 (%)	2021 (%)	AAPC	Valor de p	1990 (%)	2021 (%)	AAPC	Valor de p	1990 (%)	2021 (%)	AAPC	Valor de p
Brasil	23,3	15,2	-1,3	< 0,001	18,9	12,8	-1,2	< 0,001	27,8	18,0	-1,4	< 0,001
Quintil												
Baixo	17,7	15,2	-0,5	< 0,001	14,8	12,9	-0,5	< 0,001	20,6	17,9	-0,4	< 0,001
Baixo-médio	18,1	14,9	-0,6	< 0,001	15,6	12,7	-0,7	< 0,001	20,8	17,4	-0,5	< 0,001
Médio	20,2	13,3	-1,3	< 0,001	17,6	11,3	-1,4	< 0,001	22,5	15,3	-1,2	< 0,001
Médio-alto	22,9	14,3	-1,5	< 0,001	18,9	12,0	-1,4	< 0,001	27,0	16,8	-1,7	< 0,001
Alto	26,4	15,8	-1,6	< 0,001	20,9	13,1	-1,5	< 0,001	32,2	18,9	-1,7	< 0,001

A Tabela 1 aponta que o sexo masculino apresentou maior probabilidade de morte em todo o período, em comparação às mulheres. Ocorreu declínio da mortalidade por DCNT segundo sexo e em todos os quintis em toda a série histórica, sendo a probabilidade de morte masculina aproximadamente 47% (1990) maior que a feminina, e 40% (2021) para o total de DCNT. Em mulheres, a mortalidade passou de 18,9% para 12,8% (AAPC = -1,2; p < 0,001), e em homens, de 27,8% para 18% (AAPC = -1,4; p < 0,001). Nos quintis de pior SDI, os declínios foram menores em ambos os sexos. Entre as mulheres: quintil baixo (AAPC = -0,5; p < 0,001) e médio-baixo (AAPC = -0,7; p < 0,001). Entre os homens (AAPC = -0,4; p < 0,001) e médio-baixo (AAPC = -0,5; p < 0,001). Nos demais quintis, o AAPC variou entre -1,2 e -1,7, sendo a maior a redução observada quintil de alto SDI em ambos os sexos. Ressalta-se que o declínio da mortalidade total por DCNT foi mais acentuado nas UFs pertencentes ao quintil de SDI alto, com uma redução anual de 1,6% em comparação às UFs com SDI baixo, cuja redução média foi de 0,5% ao ano (Tabela 1).

Para o Brasil, observou-se declínio estatisticamente significativo na probabilidade de morte prematura por DCNT nos períodos de 1990 a 1996 (APC = -0,5; p = 0,050), 1996 a 2015 (APC = -1,8; p < 0,001) e 2015 a 2021 (APC = -0,9; p = 0,050), sugerindo continuidade da tendência de queda ao longo de três décadas, ainda que com variações na intensidade da redução. Entre mulheres, ocorreu declínio entre 2014 e 2019. Entre homens, em todo o período (Tabela 2).

**Tabela 2:** Variação percentual anual (APC, acrônimo em inglês) da probabilidade incondicional de morte prematura por doenças crônicas não transmissíveis, por sexo e quintil do índice sociodemográfico (SDI, acrônimo em inglês) no Brasil (1990-2021).

	Total			Feminino			Masculino		
	Período	APC	Valor de p	Período	APC	Valor de p	Período	APC	Valor de p
Brasil	1990-1996	-0,5	0,05	1990-1996	0,3	0,248	1990-1998	-0,8	0,002
	1996-2015	-1,8	< 0,001	1996-2014	0,0	0,05	1998-2014	-1,7	0,001
	2015-2021	-0,9	0,05	2014-2019	-0,6	< 0,001	2014-2021	-1,2	0,014
				2019-2021	1,9	0,23			
Quintil									
Baixo	1990-1999	-0,5	0,003	1990-2002	-0,9	0,003	1990-1999	-1,4	0,01
	1999-2002	2,3	0,003	2002-2012	-1,8	0,01	1999-2002	2,8	0,035
	2002-2006	-1,6	0,004	2012-2021	-0,7	0,12	2002-2007	-0,3	0,29
	2006-2011	0,9	0,011				2007-2011	3,3	0,043
	2011-2021	-1,5	0,001				2011-2021	-1,9	0,002
Baixo-médio	1990-2006	0,0	0,496	1990-2002	0,2	0,031	1990-1996	-0,7	0,014
	2006-2021	-1,3	< 0,001	2002-2011	-1,0	0,003	1996-2000	1,2	0,015
				2011-2014	-3,2	< 0,001	2000-2008	-0,2	0,192
				2014-2021	-0,7	0,054	2008-2021	-1,2	0,003
Médio	1990-1994	0,3	0,507	1990-1994	0,6	0,264	1990-2000	-0,9	0,066
	1994-2007	-1,8	< 0,001	1994-2009	-1,9	0,004	2000-2006	-2,2	0,017
	2007-2019	-0,6	0,046	2009-2019	-0,9	0,14	2006-2019	-0,3	0,222
	2019-2021	-5,1	< 0,001	2019-2021	-3,7	< 0,001	2019-2021	-6,0	< 0,001
Médio-alto	1990-1992	0,5	0,863	1990-1993	-0,1	0,762	1990-2021	-1,7	< 0,001
	1992-2018	-1,8	< 0,001	1993-2018	-1,9	< 0,001			
	2018-2021	0,1	0,922	2018-2021	1,5	0,044			
Alto	1990-1996	-0,4	0,112	1990-1996	-0,3	0,198	1990-1998	-0,8	0,002
	1996-2014	-2,4	< 0,001	1996-2007	-2,6	< 0,001	1998-2014	-2,5	< 0,001
	2014-2021	-0,6	0,014	2007-2018	-1,7	< 0,001	2014-2021	-0,7	0,038
				2018-2021	1,1	0,055			

No quintil de baixo SDI, foi observado aumento da probabilidade de morte prematura por DCNT em 2,3% ao ano entre 1999-2002, reduziu por um breve período e voltou a aumentar entre 2006-2011 em 0,9% ao ano, reduzindo na década final (2011-2021) em -1,5% ao ano. Entre homens, o comportamento foi semelhante. O quintil de médio-baixo SDI manteve-se estável até 2006, com declínio em seguida de -1,3% ao ano. O quintil médio teve aumento da morte prematura até 1994, e declínio nos anos seguintes, sendo mais pronunciado entre 2019-2021 (-5,1% ao ano). O quintil médio-alto reduziu entre 1992 e 2018 (-1,8% ao ano), se estabilizando após. O quintil de alto SDI reduziu entre 1996 e 2014 (-2,4% ao ano), sendo o declínio menos pronunciado entre 2014-2021 (-0,6% ao ano). Entre mulheres, ocorreu aumento entre 2018 e 2021 (1,1% ao ano) e declínio nos demais períodos, e entre os homens, houve declínio em todos os períodos, sendo menos pronunciado entre 2014-2021 (-0,7% ao ano).

**Figura 3:**
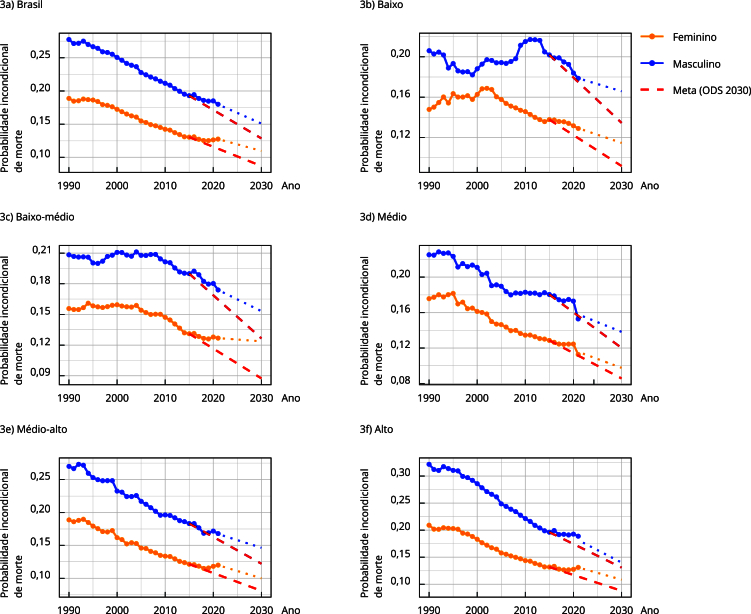
Probabilidade incondicional de morte prematura por doenças crônicas não transmissíveis, segundo sexo e quintil do índice sociodemográfico (SDI, acrônimo em inglês) no Brasil. Mensurada de 1990 a 2021, e projetada para o atingimento da meta dos Objetivos do Desenvolvimento Sustentável (ODS) de 2022-2030.

A Figura 3 apresenta as séries temporais da probabilidade incondicional de morte prematura por DCNT entre 1990 e 2021, com projeções até 2030, estratificadas por sexo e quintis do SDI. Observou-se tendência geral de declínio do indicador em todos os grupos, com maior magnitude de redução entre os homens. A análise das projeções revela que a meta de redução de 1/3 da mortalidade prematura por DCNT até 2030 provavelmente não será alcançada na população geral, nem entre os estratos por sexo ou quintil do SDI. Os maiores desvios absolutos em relação à meta concentram-se, em geral, nos quintis de menor SDI, especialmente o quintil baixo, onde os valores observados apresentaram maior instabilidade ao longo do período e redução mais recente e menos acentuada. Quanto ao sexo, não se observou padrão uniforme na diferença entre os valores projetados e a meta: em alguns quintis, como o médio-baixo e o alto, o distanciamento em relação à meta foi ligeiramente maior entre as mulheres. O padrão temporal observado sugere a persistência de desigualdades no risco de morte prematura por DCNT, com tendência à convergência apenas parcial entre os estratos socioeconômicos até 2030.

Nota: a linha tracejada em vermelho representa a trajetória hipotética da probabilidade incondicional de morte que seria necessária a cada ano para que os estados atingissem a meta estabelecida pelos ODS até 2030.

## Discussão

O estudo apontou a redução da probabilidade de morte prematura por DCNT entre 1990 e 2021 no Brasil por sexo e em todos os quintis de SDI. Os homens apresentaram probabilidade de morte mais elevada comparando-se às mulheres.

Ao analisar as projeções para 2030, a meta de reduzir a mortalidade prematura por DCNT em 1/3 não será atingida em nenhum cenário, independentemente do sexo ou do nível de desenvolvimento sociodemográfico. Observou-se que os maiores desvios em relação à meta ocorreram, em geral, nos quintis de menor SDI, especialmente o quintil baixo. No entanto, não se identificou um padrão uniforme entre homens e mulheres: em alguns quintis, as projeções indicaram maior distanciamento da meta entre as mulheres, evidenciando a complexidade do comportamento do indicador segundo sexo e contexto socioeconômico.

O indicador “probabilidade incondicional de morte prematura por DCNT” foi preconizado pela OMS para monitoramento do desempenho dos países nas ações de enfrentamento a essas doenças e seus fatores de risco. A escolha do limite inferior, de 30 anos, representa o ponto no ciclo de vida em que o risco de mortalidade para os quatro grupos de doenças crônicas estudadas começa a aumentar na maioria das populações, a partir de níveis muito baixos em idades mais jovens. O limite superior, de 70 anos, foi escolhido por identificar uma faixa etária em que essas mortes por DCNT são consideradas prematuras em quase todos os países e regiões ^22^.

No Brasil, a transição demográfica, seguida de uma transição epidemiológica acelerada, tem sido destacada em outros estudos ^33^ ^,^ ^34^. As DCNT correspondem a cerca de três quartos das mortes no país e, destas, um terço é composto por mortes prematuras ^34^, indicando a magnitude do problema. Entretanto, como indicado no estudo atual, ocorreu redução das taxas de mortalidade ^27^ ^,^ ^34^ e da probabilidade de morte no período. É possível que tais decréscimos sejam resultado da melhoria das condições gerais de vida, dos cuidados em saúde, como a expansão da atenção primária, do acesso a medicamentos, melhora da assistência de média e alta complexidade, além da redução de fatores de risco como a prevalência de tabagismo e o aumento da prática de atividade física ^34^. Soma-se o fato do Sistema Único de Saúde (SUS) ter contribuído com a melhoria de vários indicadores e resultados de saúde, pautado pelos princípios de universalidade, equidade, integralidade, descentralização e envolvimento comunitário ^35^. No entanto, este declínio não foi constante no tempo. Estudos apontam que, entre 2016 e 2021, ocorreram aumentos nas taxas de mortalidade, atribuídos às desigualdades socioeconômicas, medidas de austeridade fiscal e subfinanciamento do SUS ^20^ ^,^ ^36^ ^,^ ^37^. Além disso, foi observado que, em 2021, com o advento da pandemia de COVID-19, ocorreu a piora dos estilos de vida, e a associação com a COVID-19 resultou no aumento da mortalidade por DCNT ^18^ ^,^ ^19^ ^,^ ^38^. A piora dos indicadores relacionados às doenças cardiovasculares foi evidenciada pelo crescimento das mortes ocorridas nos domicílios, em áreas vulneráveis ^39^ ^,^ ^40^. Tal observação pode se justificar tanto pela redução do acompanhamento e monitoramento dos pacientes com DCNT no período da pandemia, quanto pela COVID-19 longa, que pode ter impactado o aumento das taxas de mortalidade por DCNT no período pós-pandemia ^41^. 

Cabe destacar que os efeitos adversos observados no período recente não podem ser atribuídos exclusivamente à pandemia de COVID-19. A partir de 2016, o país vivenciou um enfraquecimento das políticas públicas de controle do tabagismo, marcado pela estagnação da política de preços e impostos sobre produtos derivados do tabaco, e por crescente interferência da indústria no ambiente político e jurídico, caracterizando-se a influência dos determinantes comerciais em saúde ^11^. Tais retrocessos comprometeram a ampliação de medidas regulatórias e ações de cessação, com efeitos particularmente marcantes entre as mulheres e em grupos de maior renda. Evidências indicam que os riscos atribuíveis ao tabagismo em mulheres, especialmente para doenças cardiovasculares e diabetes, podem ser superiores aos observados entre homens, e que o tabagismo está causalmente associado ao diabetes tipo 2. Esses fatores podem ter contribuído para a estagnação ou mesmo reversão das tendências favoráveis entre determinados estratos, como observado no presente estudo ^42^ ^,^ ^43^. Tais retrocessos refletem a influência dos determinantes comerciais da saúde que dificultam a governança sanitária e a consolidação de políticas públicas voltadas à prevenção dos fatores de risco para DCNT. Nesse contexto, o avanço da reforma tributária em curso no país representa uma oportunidade estratégica para fortalecer medidas fiscais sobre produtos nocivos à saúde, especialmente tabaco, bebidas alcoólicas e alimentos ultraprocessados, com potencial de impacto não apenas sobre as projeções de mortalidade até 2030, mas também sobre a sustentabilidade da prevenção de DCNT no longo prazo.

Entre 1990 e 2021, o Brasil registrou importantes avanços na prevenção e controle dos fatores de risco para DCNT, os quais possivelmente contribuíram para o declínio da mortalidade prematura observado no período. Destacam-se, nesse contexto, a implementação de políticas públicas voltadas ao controle do tabagismo − como a criação da Política Nacional de Controle do Tabaco, as restrições à propaganda de produtos derivados do tabaco e a taxação progressiva −, que resultaram na redução expressiva da prevalência do tabagismo entre adultos ^43^ ^,^ ^44^. Adicionalmente, políticas intersetoriais, como o Programa Saúde na Escola, as ações de vigilância e promoção da alimentação saudável, e a regulamentação da rotulagem de alimentos ultraprocessados, buscaram atuar sobre os determinantes comportamentais das DCNT ^45^ ^,^ ^46^ ^,^ ^47^. Apesar desses avanços, persistem desafios significativos, especialmente no enfrentamento à obesidade, ao consumo nocivo de álcool e à inatividade física, cujas prevalências vêm se mantendo elevadas ou em crescimento nas últimas décadas ^47^. Além disso, as desigualdades regionais e socioeconômicas limitam o impacto dessas políticas em grupos mais vulneráveis, comprometendo a equidade dos resultados em saúde. O fortalecimento de ações regulatórias, o enfrentamento dos determinantes comerciais da saúde e a proteção das políticas públicas contra interesses econômicos adversos permanecem como desafios centrais para alcançar as metas estabelecidas até 2030 ^48^.

Destaca-se a diferença do indicador probabilidade de morte prematura por DCNT quanto ao sexo, sempre mais elevado entre os homens. Outros estudos também apontaram esta diferença. Em 2010, a magnitude deste indicador foi de cerca de 22,8% para os homens e 15,4% para as mulheres ^49^. Uma das explicações para este fato é a maior prevalência dos fatores de risco entre homens, especialmente o tabagismo, consumo de álcool e hipertensão arterial sistêmica ^50^. Também pode haver relação com as maiores exposições ocupacionais entre homens ^51^. Além disso, os homens procuram menos os serviços de saúde, o que pode resultar em atraso no diagnóstico e tratamento de doenças como infarto agudo do miocárdio, acidente vascular cerebral e câncer, que causam taxas elevadas de mortalidade ^49^.

Em relação às projeções para 2030, embora as taxas de mortalidade por DCNT estejam reduzindo ao longo dos anos, houve períodos de aumento em 2016 e 2021. Adicionalmente, cabe mencionar que as projeções para 2030 apontam para a possibilidade de não atingimento da meta dos ODS para essas doenças. É necessário manter o monitoramento contínuo para verificar possíveis mudanças ao longo dos próximos anos.

Análises anteriores também apontaram que, entre os anos 1990 e 2016, o Brasil observou um decréscimo de 34% nas taxas de mortalidade por todas as causas de morte (de 1.116,6 para 737,0 óbitos por 100 mil habitantes), porém com importantes variações entre suas UF. Os menores decréscimos foram verificados nas regiões Norte e Nordeste ^52^. Também, no estudo atual, observamos diferentes desempenhos entre as regiões. Os estados das regiões Norte e Nordeste, em sua maioria aqui representados nos quintis de baixo e baixo-médio SDI, tiveram no início da série histórica (1990) menor probabilidade de morte prematura por DCNT e mantiveram ou aumentaram a probabilidade de morte nos anos iniciais. O quintil de baixo SDI apresentou aumento da probabilidade nos anos iniciais até 2011, e declinou apenas na última década do estudo. Também o quintil de baixo-médio SDI aumentou a probabilidade nos anos iniciais do estudo. A menor probabilidade de morte por DCNT e o aumento das taxas nos anos iniciais nos quintis de pior desenvolvimento podem ser explicados por vários aspectos: piores condições socioeconômicas, menor acesso a serviços de saúde e diagnóstico ^52^, as transições demográficas e epidemiológicas, a tripla carga de doenças nessas localidades ^33^, os riscos competitivos de morte e também pela melhoria dos registros de mortalidade, captando os eventos de forma crescente nestas regiões ^28^. 

A transição demográfica refere-se à mudança do padrão de altas para baixas taxas de natalidade e de mortalidade ^53^. Assim, as transições demográficas também transcorrem com rápida transição epidemiológica, o que resulta em forte declínio das enfermidades infecciosas e aumento das DCNT ^31^ ^,^ ^33^ ^,^ ^45^ ^,^ ^46^. Em países em desenvolvimento, observa-se mais frequentemente a concomitância dos três grupos de causas de morte, configurando a tripla carga de doenças ^35^ ^,^ ^48^ ^,^ ^54^ ^,^ ^55^. Este comportamento é dito de risco competitivo, ou seja, um evento cuja ocorrência impede a observação ou, fundamentalmente, altera a probabilidade de ocorrência de um outro evento de interesse ^49^ ^,^ ^56^. Ou seja, a probabilidade de morrer na infância por doença infecciosa ou quando adulto jovem por causa externa reduz a probabilidade de morrer na fase adulta avançada por DCNT ^49^ ^,^ ^56^. Além disto, nos quintis de baixo e médio-baixo SDI, o aumento das mortes por DCNT também pode ser atribuído à melhoria da captação de óbitos nessas regiões ^57^.

Além das transições demográficas e epidemiológicas já descritas, é importante destacar uma mudança recente no perfil das causas de morte prematura por DCNT no Brasil. Estudo populacional de Rache et al. ^58^ identificou que, a partir dos anos mais recentes, observa-se uma transição progressiva da predominância das doenças cardiovasculares para as neoplasias como a principal causa de mortalidade prematura. Essa inflexão é particularmente evidente entre mulheres e em regiões de maior desenvolvimento socioeconômico, refletindo mudanças nos padrões de exposição aos fatores de risco, nos avanços terapêuticos e nos sistemas de detecção precoce ^58^. Embora o presente estudo tenha analisado as DCNT de forma agregada, reconhecer essa transição contribui para a compreensão das dinâmicas internas ao grupo das DCNT e reforça a importância de estratégias específicas de prevenção e controle ajustadas à composição etária, regional e de sexo da população.

Cabe considerar que o aumento da probabilidade de morte por DCNT observado nos anos iniciais da série histórica, sobretudo, entre as regiões de baixo e médio-baixo SDI, pode refletir, ao menos parcialmente, uma melhoria progressiva na capacidade de diagnóstico e registro das causas básicas de morte ^59^. A expansão da cobertura da atenção primária, a incorporação de tecnologias diagnósticas e o fortalecimento dos sistemas de informação em saúde contribuíram para a substituição de causas inespecíficas por diagnósticos mais precisos ^60^. Essa hipótese é consistente com a literatura que aponta a redistribuição de causas mal definidas como fator relevante para o aumento das estimativas de mortalidade por causas específicas em contextos historicamente marcados pelo subregistro ^61^.

No estudo atual, observam-se diferentes perfis de transição epidemiológica dependendo da condição socioeconômica. No período inicial, as mortes por DCNT foram mais elevadas nas regiões mais ricas (elevado SDI), e baixa mortalidade por DCNT nas regiões de baixo e baixo-médio SDI. Entretanto, o declínio da mortalidade por DCNT foi mais expressivo em todo o período nas regiões ricas, cerca de três vezes mais elevada quando comparada ao baixo SDI. No quintil de baixo SDI ocorreu estabilidade e aumento de mortalidade até 2011, quando então reduziu. Ao final do período do presente estudo, o quintil de baixo SDI tendeu a apresentar convergência na probabilidade de morte em relação aos demais quintis de SDI. 

Estas diferenças, em especial no período inicial, podem ser explicadas, em parte, pelas desigualdades sociais, diferenças culturais e distintas formas de organização e de capacidade dos sistemas regionais e locais de saúde ^29^, bem como pelo acesso desigual a serviços e diagnósticos. Além disso, é importante considerar que os diferentes patamares de mortalidade por DCNT e os declínios heterogêneos observados entre os quintis do SDI também podem refletir padrões distintos de exposição aos fatores de risco. Indivíduos residentes em regiões com menor SDI tendem a apresentar maiores prevalências de tabagismo, consumo nocivo de álcool, inatividade física, alimentação inadequada e obesidade, em razão de condições estruturais adversas, como menor renda, baixa escolaridade, precariedade habitacional e escassez de ambientes promotores de saúde ^29^. Dessa forma, o *status* socioeconômico atua não apenas como fator determinante do acesso à saúde, mas também como mediador da exposição aos fatores de risco, contribuindo de modo significativo para a configuração das desigualdades na mortalidade prematura por DCNT entre os quintis analisados.

Uma possível explicação para o aumento recente da probabilidade de morte prematura por DCNT entre mulheres no quintil médio-alto de SDI, observado entre 2018 e 2021, pode estar relacionada a mudanças na prevalência de fatores de risco comportamentais, notadamente o tabagismo ^44^. Embora o Brasil tenha alcançado expressivas reduções na prevalência do tabagismo nas últimas décadas, evidências apontam que, a partir de 2016, houve um enfraquecimento da implementação de medidas regulatórias voltadas à cessação, especialmente entre grupos de maior escolaridade e renda. Estudos também indicam que os riscos atribuíveis ao tabagismo em mulheres, particularmente para doenças cardiovasculares e diabetes, podem ser superiores aos observados entre homens ^42^ ^,^ ^62^. Além disso, o tabagismo está causalmente associado ao desenvolvimento do diabetes tipo 2, ampliando o risco de desfechos fatais ^63^. Assim, ainda que a epidemia do tabaco no Brasil seja historicamente mais concentrada nas camadas socioeconômicas mais baixas, alterações nas políticas de controle do tabagismo e mudanças nos padrões de cessação por sexo e classe social podem ter contribuído para a estagnação ou reversão de tendências favoráveis em determinados estratos, como observado no presente estudo. A ausência de aumento concomitante nos quintis mais baixos pode estar relacionada à manutenção de políticas locais, diferenças na dinâmica do uso de tabaco ou à interação com outros fatores concorrentes de risco ou proteção.

Dentre os limites do estudo, embora o GBD realize correções por subregistro e redistribuições por códigos *garbage*, ainda assim, as correções podem não ter sido suficientes para a mensuração de dados fidedignos de mortalidade em todos os estados, em especial, entre aqueles com baixo SDI, principalmente nos primeiros anos da série histórica.

Um aspecto que merece reflexão diz respeito ao desafio de estabelecer metas globais de redução da mortalidade prematura por DCNT em contextos marcados por desigualdades estruturais profundas. Países e regiões em diferentes estágios de desenvolvimento econômico e de consolidação dos sistemas de saúde enfrentam realidades contrastantes quanto à capacidade de implementação de políticas públicas, vigilância epidemiológica e acesso à atenção integral. Esses contrastes limitam a comparabilidade direta entre regiões e comprometem, em certa medida, a viabilidade de metas uniformes. Os resultados do presente estudo ilustram esse cenário ao evidenciar desigualdades regionais persistentes na trajetória e nas projeções da mortalidade por DCNT no Brasil. Tal constatação reforça a necessidade de que metas globais sejam acompanhadas de estratégias nacionais adaptadas às especificidades locais, com atenção especial às regiões em maior vulnerabilidade social e institucional.

Em conclusão, as projeções para 2030 indicam que o Brasil poderá não atingir a meta dos ODS de reduzir em um terço a mortalidade prematura por DCNT, o que acende um alerta para a urgência do fortalecimento das políticas públicas voltadas à prevenção e ao controle dessas doenças. Ressalta-se a necessidade de intensificar ações intersetoriais e estratégias de promoção da saúde, diagnóstico precoce, acesso a tratamento oportuno e acompanhamento contínuo, especialmente no âmbito da atenção primária à saúde. É fundamental direcionar essas iniciativas às populações em maior situação de vulnerabilidade social e econômica, considerando as desigualdades regionais e estruturais que ainda persistem no país. O enfrentamento das DCNT demanda abordagens abrangentes que integrem os determinantes sociais, ambientais e comerciais da saúde, visando à redução das iniquidades e ao avanço efetivo rumo às metas estabelecidas pela Agenda 2030.
